# Risk Factors Associated With Six-Month Mortality in Hospitalized COVID-19 Patients: A Single-Institution Study

**DOI:** 10.7759/cureus.31206

**Published:** 2022-11-07

**Authors:** Navkirat Kahlon, Jasskiran Kaur, Sishir Doddi, Cameron Burmeister, Taha Sheikh, Ziad Abuhelwa, Aya Abugharbyeh, Ragheb Assaly, William Barnett, Danae Hamouda

**Affiliations:** 1 Hematology and Medical Oncology, The University of Toledo College of Medicine and Life Sciences, Toledo, USA; 2 Internal Medicine, The University of Toledo College of Medicine and Life Sciences, Toledo, USA; 3 Rheumatology, The University of Toledo College of Medicine and Life Sciences, Toledo, USA; 4 Pulmonary/Critical Care Medicine, The University of Toledo College of Medicine and Life Sciences, Toledo, USA; 5 Biostatistics and Epidemiology, The University of Toledo Medical Center, Toledo, USA

**Keywords:** lactate dehydrogenase, 6-month mortality, elevated ldh, elevated d-dimer, covid-19 mortality

## Abstract

Background

Coronavirus disease 2019 (COVID-19) infection can vary from asymptomatic infection to multi-organ dysfunction. The most serious complication of infection with COVID-19 is death. Various comorbid conditions and inflammatory markers have been associated with an increased risk of mortality, specifically within the immediate post-infection period; however, less is known about long-term mortality outcomes.

Objectives

Our objective is to determine risk factors associated with six-month mortality in hospitalized COVID-19 patients.

Methods

This is a single-institution, retrospective study. We included patients hospitalized with COVID-19 from the University of Toledo Medical Center in Toledo, Ohio, who were admitted from March 20, 2020, to June 30, 2021. This study was approved by a biomedical institutional review board at the University of Toledo. Patients with available pre-stored blood samples for laboratory testing were included, and hospital charts were assessed up to six months from the date of a positive COVID-19 test result. Two groups were created based on the mortality outcome at six months from COVID-19 positive test results: survivors and non-survivors. The clinical variables or outcomes and laboratory values were compared between the two groups using non-parametric methods due to the small sample size and non-normality of the data. Either the Mann-Whitney U-test for continuous variables or Fisher’s exact test for categorical variables was used for statistical analysis.

Results

Lactate dehydrogenase (LDH) and D-dimer levels on admission were found to be significantly higher in non-survivors than in survivors. The median high D-dimer level in non-survivors was 5.96 micrograms/milliliter (μg/mL) (interquartile range (IQR): 3.95-11.29 μg/mL) vs 1.82 μg/mL (IQR 1.13-5.55 μg/mL) in survivors (p = 0.019). Median LDH levels were also higher in non-survivors vs survivors, i.e., 621.00 international units per liter (IU/L) (IQR 440.00-849.00 IU/L) vs 328.00 IU/L (IQR 274.00-529.00 IU/L), respectively (p = 0.032). The demographic profile, comorbidity profile, and laboratory data (typically associated with short-term mortality, inflammation, and organ dysfunction) were similar between survivors and non-survivors, except for LDH and D-dimer.

Conclusion

Higher LDH and D-dimer levels on admission were found to be associated with an increased six-month mortality rate in hospitalized COVID-19 patients. These hematologic data can serve as risk stratification tools to prevent long-term mortality outcomes and provide proactive clinical care in hospitalized COVID-19 patients.

## Introduction

The coronavirus disease 2019 (COVID-19) pandemic has undoubtedly altered the course of world history since the first cases of severe acute respiratory syndrome coronavirus 2 (SARS-CoV-2) were reported in adult patients in December 2019. The virus is a single-stranded, enveloped RNA virus that primarily infects mammalian hosts [1]. SARS-CoV-2 is highly transmissible in humans and is spread commonly via respiratory droplets. The most common symptoms include fever, cough, shortness of breath, fatigue, and nausea. Common pulmonary manifestations include acute viral pneumonia; however, COVID-19 has a wide spectrum of extra-pulmonary symptom manifestations as well, ranging from asymptomatic infection to multi-organ dysfunction [2]. The most serious complication of infection with COVID-19 is death; there have been 542 million reported cases, and COVID-19 has claimed the lives of over 6.33 million people worldwide since 2019 [3].

Patients hospitalized with COVID-19 infection have unique characteristics that can impact their short-term mortality rates and disease severity. Between 60% and 90% of hospitalized patients have other comorbidities (most commonly hypertension, diabetes mellitus, cardiovascular disease (CVD), and chronic obstructive pulmonary disease), and approximately 35% of hospitalized patients experience severe disease necessitating intensive care unit level of care [1]. Symptoms, organ dysfunction, and/or psychological damage may persist for weeks to months after infection with COVID-19 in a minor subset of patients; long-term COVID-19 is a distressing complication for some after acute infection.

The pathophysiology, disease course, and validated treatment modalities have been extensively examined since the advent of the worldwide COVID-19 pandemic three years ago; however, post-acute infection data continues to surface, which dynamically shapes our knowledge of the impact COVID-19 has on patients and communities. Laboratory data obtained on hospitalized patients during acute COVID-19 infection may be a useful tool to predict post-acute mortality risk. Much of the study data relating to post-acute COVID-19 infection morbidity and mortality risk in the United States (US) is 90-day data; however, some European studies have followed patients for six months post-infection [4]. The purpose of this study is to retrospectively examine single-center data from hospitalized adult patients suffering from COVID-19 infection in Toledo, Ohio, USA, between March 2020 and June 2021 to determine the demographic, social, and laboratory factors that may be associated with a higher six-month mortality rate.

## Materials and methods

Study participants and design

Patients aged ≥18 years old with confirmed positive polymerase chain reaction (PCR) testing for COVID-19 infection, hospitalization for COVID-19 infection during the period of March 20, 2020 to June 30, 2021, and availability of adequate and viable pre-stored plasma and/or serum samples from admission (collected within two days of admission) for laboratory testing were included in the study. The number of inpatient COVID-19 cases had decreased for a brief period around June 2021, so the patient list just up to this period was chosen for the study. A list of patients hospitalized with COVID-19 during this period was obtained from the information technology department and pulmonary medicine team at the University of Toledo Medical Center. The medical records of patients who tested positive for COVID-19 were reviewed until six months after the date of the positive COVID-19 test. Only de-identified data has been reported. Two primary groups were created based on the mortality outcome of COVID-19 patients at six months from the COVID-19 positive test: survivors and non-survivors. The final analysis included twenty-eight patients, seventeen survivors, and eleven non-survivors.

Statistical analysis

The primary objective of this study is to identify risk factors that may be associated with six-month mortality. The clinical variables or outcomes and laboratory values were compared using non-parametric methods due to the small sample size and non-normality of the data. Either the Mann-Whitney U-test for continuous variables or Fisher’s exact test for categorical variables was used for statistical analysis. All analyses were performed using R 4.0.3 (R Foundation for Statistical Computing, Vienna, Austria) and an alpha level of 0.05 was considered statistically significant.

Ethical approval

The approval for our retrospective study conducted at the University of Toledo Medical Center was granted by the biomedical Institutional Review Board (IRB) prior to beginning the collection of data, IRB number 300722-UT. 

## Results

Our final analysis included a total of 28 patients. Eleven of the 28 were non-survivors and seventeen of the 28 were survivors six months after hospitalization for COVID-19 infection. No difference was found in the demographic profile of patients in the two groups. The median age, sex, and ethnicity were statistically similar between the two groups. The median age was 64 years (interquartile range (IQR): 59.00-67.00 years) for non-survivors and 61 years (IQR: 53.00-68.00 years) for survivors (p = 0.524). There was no statistically significant difference in sex distribution or classification by the racial and ethnic group either. About 45.5% of non-survivors were male, while 76.5% of survivors were male (p = 0.125). Among non-survivors, 36.4% were White, 54.5% were Black, and 9.1% were Hispanic; among survivors, 47.1% were White, 41.2% were Black, and 0% were Hispanic (p = 0.486). No statistically significant difference in six-month mortality outcome was found based on the selected comorbidity profile: coronary artery disease, atrial fibrillation, cerebrovascular accidents, peripheral artery disease, cancer, and smoking.

In non-survivor and survivor groups, the past medical history of coronary artery disease was 18.2% and 29.4% (p = 0.668), atrial fibrillation was 9.1% and 35.3 % (p = 0.191), the cerebrovascular accident was 36.4% and 23.5% (p = 0.672), the peripheral arterial disease was 18.2% and 29.4% (p = 0.668), cancer was 9.1% and 29.4% (p = 0.355), and smoking was 63.6% and 47.1% (p = 0.460), respectively. Intubation rates and hemodialysis rates during hospitalization for COVID-19 were similar among the two groups. Intubation was required in 81.8% of non-survivors and 47.1% of survivors (p = 0.115), and hemodialysis was required in 36.4% of non-survivors and 5.9% of survivors (p = 0.062). The rehospitalization rates were also similar between the two groups, i.e., 18.2% and 35.3% in non-survivors and survivors, respectively (p = 0.115). These results are summarized in Table 1.

**Table 1:** Demographics and comorbidities at presentation in hospitalized COVID-19 patients and six-month mortality outcome. IQR: interquartile range; M: male; %: percent; CAD: coronary artery disease; Y: yes; Afib: atrial fibrillation; CVA: cerebrovascular accident; PAD: peripheral artery disease; vent: ventilator, HD/CVVHD: need for hemodialysis/continuous venovenous dialysis during hospitalization for COVID-19 infection.

	Non-survivors	Survivors	p-value
Number of patients	11	17	
Age (median (IQR))	64.00 (59.00, 67.00)	61.00 (53.00, 68.00)	0.524
Sex = M (%)	5 (45.5)	13 (76.5)	0.125
Ethnicity			0.486
White	4 (36.4)	8 (47.1)	
Black	6 (54.5)	7 (41.2)	
Hispanic	1 (9.1)	0 (0.0)	
Unknown	0 (0.0)	2 (11.8)	
CAD = Y (%)	2 (18.2)	5 (29.4)	0.668
Afib = Y (%)	1 (9.1)	6 (35.3)	0.191
CVA = Y (%)	4 (36.4)	4 (23.5)	0.672
PAD = Y (%)	2 (18.2)	5 (29.4)	0.668
Cancer = Y (%)	1 (9.1)	5 (29.4)	0.355
Smoking = Y (%)	7 (63.6)	8 (47.1)	0.460
Vent = Y (%)	9 (81.8)	8 (47.1)	0.115
HD/CVVHD = Y (%)	4 (36.4)	1 (5.9)	0.062
Rehospitalization (%)			0.115
None	9 (81.8)	11 (64.7)	
Once	0 (0.0)	5 (29.4)	
Twice	2 (18.2)	1 (5.9)	

Laboratory data at presentation in hospitalized COVID-19 patients were compared between non-survivors and survivors. Between the two groups, no significant difference in either the highest hemoglobin (Hgb) or lowest Hgb levels was observed. The median highest Hgb level was 12.10 grams per deciliter (g/dl) (IQR 10.70-13.40 g/dl) in non-survivors in comparison to 12.40 g/dl (IQR 10.50-13.60 g/dl) in survivors (p = 0.981). The lowest median Hgb level was 9.50 g/dl (IQR 7.20-12.00 g/dl) in non-survivors in comparison to 8.20 g/dl (IQR 6.90-9.60 g/dl) in survivors (p = 0.346). The highest median white blood cell (WBC) count and the lowest median WBC were also similar. The median high WBC level was 19.70 grams per deciliter (g/dl) (IQR 14.87-22.81 g/dl) in non-survivors in comparison to 15.06 g/dl (IQR 11.85-18.86 g/dl) in survivors (p = 0.269). The low median WBC level was 4.70 g/dl (IQR 4.70-7.20g/dl) in non-survivors in comparison to 7.07 g/dl (IQR 4.47-8.40 g/dl) in survivors (p = 0.359). There was no difference in median high and median low platelets (plt) between the two groups. The median high plt level was 354.00 × 1000 cells per microliter of blood (cells/µl) (IQR 200.50-457.50 × 1000 cells/µl) in non-survivors in comparison to 348.00 × 1000 cells/µl (IQR 288.00-368.00 × 1000 cells/µl) in survivors (p = 0.944). The median low plt level was 146.00 × 1000 cells/µl (IQR 93.50-200.50 × 1000 cells/µl) in non-survivors in comparison to 151.00 × 1000 cells/µl (IQR 112.00-195.00 × 1000 cells/µl) in survivors (p = 0.510). The median of low absolute neutrophil count (ANC) was also similar between the two groups, i.e., 2.80 × 1000 cells per cubic millimeter (cells/mm^3^) (IQR 2.00-7.85 × 1000 cells/mm^3^) vs 4.05 × 1000 cells/mm^3^ (IQR 2.12-5.68 × 1000 cells/mm^3^) in non-survivors and survivors, respectively (p = 0.869). The median of low absolute lymphocyte count (ALC) was similar between the two groups as well, i.e., 0.60 × 1000/ mm^3^ (IQR 0.40-1.05 × 1000 cells/mm^3^) vs 0.75 × 1000 cells/mm^3^ (IQR 0.32-1.15 × 1000 cells/mm^3^) in non-survivors and survivors, respectively (p = 0.912). The median peak bilirubin level was similar between the two groups, i.e., 0.50 milligrams per deciliter (mg/dl) (IQR 0.40-0.80 mg/dl) vs 0.60 mg/dl (IQR 0.40-0.85 mg/dl) in non-survivors and survivors, respectively (p = 0.783). The median low albumin level was also statistically similar between the non-survivors and survivors, i.e., 2.70 grams per deciliter (g/dl) (IQR 2.20-3.30 g/dl) vs 2.85 g/dl (IQR 2.38-3.10 g/dl), respectively (p = 0.843). 

Inflammatory markers such as median high D-dimer and median lactate dehydrogenase (LDH) were higher in non-survivors compared to survivors. The median high D-dimer level in non-survivors was 5.96 micrograms/milliliter (μg/mL) (IQR 3.95-11.29 μg/mL) vs 1.82 μg/mL (IQR 1.13-5.55 μg/mL) in survivors (p = 0.019). Median LDH levels were also higher in non-survivors compared to survivors, i.e., 621.00 international units per liter (IU/L) (IQR 440.00-849.00 IU/L) vs 328.00 IU/L (IQR 274.00, 529.00 IU/L), respectively (p = 0.032). Other inflammatory marker levels, such as high and low median ferritin, median erythrocyte segmentation rate (ESR), and high and low median C-reactive protein (CRP), were similar between the two groups. The median high ferritin level was 1499.00 micrograms/liter (µg/L) (IQR 660.00-2425.00 µg/L) in non-survivors in comparison to 793.00 μg/L (IQR 385.50-2723.50 μg/L) in survivors (p = 0.775). The median low ferritin level nadir was 706.00 µg/L (IQR 325.00-989.50 µg/L) in non-survivors in comparison to 406.00 μg/L (IQR 248.00-1267.50 μg/L) in survivors (p = 0.586). The median ESR was 80.00 millimeters/hour (mm/hr) (IQR 61.50-97.75 mm/hr) and 48.50 mm/hr (IQR 26.00-93.00 mm/hr) in non-survivors and survivors, respectively. The median low CRP level was statistically similar among non-survivors and survivors, i.e., 78.10 mg/dL (IQR 27.75-164.00 mg/dL) and 30.70 mg/dL (IQR 21.90-69.35 mg/dL), respectively (p = 0.223). The median high CRP in non-survivors was 197.00 mg/dL (IQR 140.50-299.00 mg/dL) and 142.00 mg/dL (IQR 102.45-229.00 mg/dL) in survivors (p = 0.299). The creatinine peak was also similar between the two groups. The median creatinine level peak was 3.68 mg/dL (IQR 1.13-5.74 mg/dL) in non-survivors in comparison to 1.35 mg/dL (IQR 1.23-3.66 mg/dL) in survivors (p = 0.239). These results are summarized in Table 2.

**Table 2:** Laboratory data at initial hospitalization in hospitalized COVID-19 patients and six-month mortality outcome. Hgb: hemoglobin in grams per deciliter (g/dl); IQR: interquartile range; WBC: white blood cell count × 1000 in cells per cubic millimeter of blood; PLT: platelets count × 1000 cells per microliter of blood.; ANC: absolute neutrophil count per cubic millimeter of blood; ALC: absolute lymphocyte count per cubic millimeter of blood; bili: bilirubin in milligrams per deciliter (mg/dl); Alb: albumin grams per deciliter (g/dL); ESR: erythrocyte sedimentation rate in millimeters/hour; CRP: C-reactive protein in milligrams/deciliter; LDH: lactate dehydrogenase in international units per liter (IU/L); Cr: creatinine in milligrams/deciliter. ⁺Statistically significant.

	Non-survivor	Survivor	p-value
Number of patients	11	17	NA
Peak Hgb (median (IQR))	12.10 (10.70, 13.40)	12.40 (10.50, 13.60)	0.981
Hgb nadir (median (IQR))	9.50 (7.20, 12.00)	8.20 (6.90, 9.60)	0.346
WBC nadir (median (IQR))	4.70 (4.08, 7.20)	7.07 (4.47, 8.40)	0.359
Peak WBC (median (IQR))	19.70 (14.87, 22.81)	15.06 (11.85, 18.86)	0.269
Peak PLT (median (IQR))	354.00 (200.50, 457.50)	348.00 (288.00, 368.00)	0.944
Low PLT (median (IQR))	146.00 (93.50, 200.50)	151.00 (112.00, 195.00)	0.510
Low ANC (median (IQR))	2.80 (2.00, 7.85)	4.05 (2.12, 5.68)	0.869
Low ALC (median (IQR))	0.60 (0.40, 1.05)	0.75 (0.32, 1.15)	0.912
Peak bili (median (IQR))	0.50 (0.40, 0.80)	0.60 (0.40, 0.85)	0.783
Alb nadir (median (IQR))	2.70 (2.20, 3.30)	2.85 (2.38, 3.10­)	0.843
Peak ESR (median (IQR))	80.00 (61.50, 97.75)	48.50 (26.00, 93.00)	0.233
Peak D-dimer (median (IQR))	5.96 (3.95, 11.29)	1.82 (1.13, 5.55)	0.019⁺
Peak ferritin (median (IQR))	1499.00 (660.50, 2425.00)	793.00 (385.50, 2723.50)	0.775
Ferritin nadir (median (IQR))	706.00 (325.00, 989.50)	406.00 (248.00, 1267.50)	0.586
CRP nadir (median (IQR))	78.10 (27.75, 164.00)	30.70 (21.90, 69.35)	0.223
Peak CRP (median (IQR))	197.00 (140.50, 299.00)	142.00 (102.45, 229.00)	0.299
Peak LDH (median (IQR))	621.00 (440.00, 849.00)	328.00 (274.00, 529.00)	0.032⁺
Peak Cr (median (IQR))	3.68 (1.13, 5.74)	1.35 (1.23, 3.66)	0.239

## Discussion

To our knowledge, this is the first study in the United States analyzing risk factors associated with six-month mortality in hospitalized COVID-19 patients though some literature from European countries exists in this context [4,5]. Studies on the predictors of acute-mortality outcomes in patients with COVID-19 have been published in the US, but not in the context of six-month mortality. Our study found higher median lactate dehydrogenase (LDH) and D-dimer levels on initial hospitalization were associated with six-month mortality in hospitalized patients with COVID-19 infection. Our results found that the median D-dimer in the non-survivor group was 3.3 times higher than that of the survivor group (alive at six months). The median LDH in the non-survivor group was approximately 1.9 times higher than that of the survivor group.

LDH is an enzyme found in many tissues in our bodies; consequently, tissue damage leads to increased LDH released into the blood. Other studies involving COVID-19 patients have found an association between higher LDH levels and poor prognostic outcomes [6-8]. Fan et al. found elevated LDH at admission in ICU patients compared to their non-ICU group (p-value <0.001) [6]. Yan et al. analyzed blood samples from 485 infected patients from Wuhan, China, to identify biomarkers of COVID-19 mortality via machine learning tools, and they found LDH, lymphocytes, and high-sensitivity CRP predicts mortality more than 10 days in advance with more than 90% accuracy [7]. Specifically, levels of LDH alone in the blood were highly predictive of COVID-19 mortality. Higher levels of LDH are used as markers of tissue damage in liver disease and lung tissue death. Furthermore, published reports suggest LDH is a prognostic indicator of lung fibrosis in severe interstitial lung disease [8]. In a study conducted in Yichang, China, Lv et al. reported higher LDH levels in non-survivors of COVID-19 than survivors in the post-COVID lung fibrosis stage. The median survival time was 27.5 days in patients with high LDH, quantified as greater than 263 IU/L, compared to those with low LDH, quantified as less than 263 IU/L, who had a median survival time of 40 days [9].

D-dimer is a degradation product of cross-linked fibrin and is often used as a marker for hemostatic abnormalities, specifically blood clots. In many studies [7], increasing D-dimer levels have been associated with COVID-19 severity as well as mortality. In a study on persistent D-dimer levels in patients with convalescent COVID-19, the observed rate of increased D-dimer levels was higher in hospitalized patients and predicted thrombotic complications [10]. In a case-control study by Yao et al., D-dimer elevation upon admission was associated with increased disease severity and in-hospital mortality [11]. Similar to our study, Li et al. concluded normal D-dimer on presentation, day one, is predictive of 28-day survival [12]. Furthermore, a normal D-dimer on day three is even more predictive of 28 days of survival. In contrast to Li et al., our study analyzed a longer time frame of six months vs 28-day survival. Previous research has shown that D-dimer values are frequently elevated in COVID-19 patients; however, they are even higher in patients with severe COVID-19 compared to those with milder forms. In a retrospective cohort study in Wuhan, China, conducted by Zhou et al., it was reported that D-dimer levels, specifically those greater than 1 ug/mL, were approximately ninefold higher in non-survivors than survivors in a 22-day mortality period [13].

We did not find significant differences between the two groups in either peak or nadir values of hematologic parameters, i.e., hemoglobin (Hgb), white blood cell (WBC), platelet count (plt), absolute neutrophil count (ANC), and absolute lymphocyte count (ALC). Anemia and thrombocytopenia have previously been found to be associated with disease severity. Thrombocytopenia has also been linked to an increased risk of COVID-19 mortality [14,15]. One of the first studies to report an association between thrombocytopenia as a risk factor for mortality in COVID-19 patients was conducted in China. Thrombocytopenia was associated with an approximately three times higher risk of mortality compared to those without thrombocytopenia. This data is in contrast with our findings and may be attributed to the difference in sample size [16]. A meta-analysis of the clinical characteristics of COVID-19 patients reported normal leukocyte counts in 69.7% of patients, lymphopenia in 59.5% of patients, and leukocytosis in 12.6% [17]. We did not report significant differences in leukocyte counts between survivors and non-survivors. The peak and nadir of median ferritin, c-reactive protein (CRP) levels, and erythrocyte sedimentation rate (ESR) were similar between the two groups. In a systematic review by Iwamura et al., ESR and CRP levels were elevated regardless of the degree of severity of the infection [18]. Our data is consistent with these findings. 

No significant association between six-month mortality and demographic characteristics such as age, sex, race, and ethnicity was found. In contrast to this data, a cohort study in Germany found the odds of six-month all-cause mortality increased by 2.21 for every 10 years of age [4]. They also report that the female sex is protective as women had improved long-term outcomes than males. The limited sample size of our study may explain the difference within our findings. Larger studies to elucidate associations between demographic characteristics and six-month mortality may be warranted. Comorbid conditions such as coronary artery disease (CAD), atrial fibrillation (Afib), cerebrovascular accidents (CVAs), and peripheral artery disease (PAD) did not predict mortality. A systemic review of predictors of mortality by Tian et al. reports cardiovascular disease and cerebrovascular disease were associated with a higher risk of mortality, even though mechanisms are not yet understood [19]. In a French study, coronary artery calcifications (CACs) were found to be an independent predictor of six-month mortality in COVID-19 patients, even those without known atherosclerotic cardiovascular disease [20]. Published data is unclear in the association of various comorbid conditions and the prediction of mortality and severity of COVID-19.

Limitations

This study has limitations. The study was conducted in one specific institution and had a limited sample size. The external validity of our results would increase with studies in various regions and institutions and with larger sample sizes. Due to the retrospective nature of the study, previously collected data was utilized, which limited the variables that were analyzed. Lastly, laboratory data were analyzed from a one time frame, from initial hospitalization; therefore, further studies are needed to elucidate possible time-dependent changes in prognostic variables.

## Conclusions

In conclusion, the results from our study provide potential risk factors at the presentation that are associated with mortality at six months. Higher levels of LDH and D-dimer were found to be associated with six-month mortality. To our knowledge, this study is the first to analyze six-month mortality in the United States. The role of these factors as prognostic and predictive markers needs to be studied further with larger sample sizes. Hematological markers are easily obtained in the hospital setting and can serve as biomarkers for rapid screening, risk stratification, and mortality prediction. Our findings, with further validation, have the potential to provide novel long-term mortality risk stratification tools to identify high-risk hospitalized COVID-19 patients and ideally prevent mortality via early intervention and triage.
